# β-lactam precision dosing in critically ill children: Current state and knowledge gaps

**DOI:** 10.3389/fphar.2022.1044683

**Published:** 2022-12-01

**Authors:** Sonya Tang Girdwood, Kathryn Pavia, Kelli Paice, H. Rhodes Hambrick, Jennifer Kaplan, Alexander A. Vinks

**Affiliations:** ^1^ Division of Clinical Pharmacology, Department of Pediatrics, Cincinnati Children’s Hospital Medical Center, Cincinnati, OH, United States; ^2^ Division of Hospital Medicine, Department of Pediatrics, Cincinnati Children’s Hospital Medical Center, Cincinnati, OH, United States; ^3^ Department of Pediatrics, University of Cincinnati College of Medicine, Cincinnati, OH, United States; ^4^ Division of Critical Care Medicine, Department of Pediatrics, Cincinnati Children’s Hospital Medical Center, Cincinnati, OH, United States; ^5^ Division of Nephrology and Hypertension, Department of Pediatrics, Cincinnati Children’s Hospital Medical Center, Cincinnati, OH, United States

**Keywords:** beta-lactam antibiotics, precision dosing, population pharmacokinetic models, critically ill, pediatrics

## Abstract

There has been emerging interest in implementing therapeutic drug monitoring and model-informed precision dosing of β-lactam antibiotics in critically ill patients, including children. Despite a position paper endorsed by multiple international societies that support these efforts in critically ill adults, implementation of β-lactam precision dosing has not been widely adopted. In this review, we highlight what is known about β-lactam antibiotic pharmacokinetics and pharmacodynamics in critically ill children. We also define the knowledge gaps that present barriers to acceptance and implementation of precision dosing of β-lactam antibiotics in critically ill children: a lack of consensus on which subpopulations would benefit most from precision dosing and the uncertainty of how precision dosing changes outcomes. We conclude with opportunities for further research to close these knowledge gaps.

## Introduction

β-Lactam antibiotics (e.g., cephalosporins, carbapenems) cover many Gram-positive and Gram-negative bacteria but demonstrate high pharmacokinetic/pharmacodynamic (PK/PD) variability in critically ill patients (Craig, 1998; Roberts and Lipman, 2009; Droege et al., 2016; Phe et al., 2020; Barreto et al., 2021). Their efficacy is dependent on time that free, non-protein-bound, concentrations are above the bacterial minimum inhibitory concentrations (*f*T > _MIC_) (Craig, 1998; Roberts and Lipman, 2009; Droege et al., 2016; Phe et al., 2020; Barreto et al., 2021). Since β-lactam concentrations are not measured routinely, clinicians often assume standard β-lactam antibiotic doses achieve exposures necessary for optimal bactericidal activity while avoiding toxicity. The lack of monitoring in children is concerning as PK variability may be higher due to maturation effect at younger ages and due to highly variable weights at older ages (Lu and Rosenbaum, 2014). It is known that standard intermittent β-lactam dosing in critically ill adult and pediatric patients leads to highly variable concentrations (up to 100-fold) (Roberts et al., 2010; Garot et al., 2011; Duszynska et al., 2012; Roberts et al., 2012; Sime et al., 2012; Kongthavonsakul et al., 2016; Shoji et al., 2016; De Cock et al., 2017; Beranger et al., 2019; Al-Shaer et al., 2020a; Abdulla et al., 2020; Tang Girdwood et al., 2020a; Lonsdale et al., 2020; Saito et al., 2020; Hartman et al., 2021; Tang Girdwood et al., 2021). This high PK variability leads to inconsistent PD target attainment, placing patients at risk of suboptimal bactericidal activity. β-lactam antibiotics have been associated with specific toxicities: ceftriaxone with hepatotoxicity, (Nakaharai et al., 2016), piperacillin/tazobactam with acute kidney injury (AKI), (Hammond et al., 2017; Mellen et al., 2017; Chen et al., 2018; Luther et al., 2018; Avedissian et al., 2020), and cefepime and meropenem with neurotoxicity (Appa et al., 2017; Imani et al., 2017; Payne et al., 2017; Roger and Louart, 2021). Data have emerged to show a relationship between high β-lactam exposure and toxicities in adults (Imani et al., 2017). Therefore, high PK variability can also place patients at risk for toxicity.

Due to the known high PK/PD variability of β-lactam antibiotics in critically ill patients, a position paper endorsed by multiple international societies recommends routine therapeutic drug monitoring (TDM) of β-lactam antibiotics in critically ill adult patients (Abdul-Aziz et al., 2020). However, β-lactam TDM remains a source of debate as others find the evidence for routine monitoring to be sparse (Dilworth et al., 2022). In this narrative review, we explore the merits of TDM for beta-lactams, specifically model-informed precision dosing (MIPD); knowledge gaps required to be filled prior to implementation of MIPD in critically ill children; and potential future directions for research of β-lactam antibiotics in pediatric critical illness. We focus specifically on ceftriaxone, piperacillin/tazobactam, cefepime and meropenem as they are the most commonly used intravenous β-lactam antibiotics used in critically ill children in the United States.

## Defining model-informed precision dosing

Much of the literature regarding β-lactam antibiotic monitoring refers to traditional TDM, (Roberts et al., 2010; Cies et al., 2018; Veiga and Paiva, 2018; Al-Shaer et al., 2020a; Abdul-Aziz et al., 2020), in which concentrations are measured at specified timepoints and compared to a population-defined target. Traditional TDM, however, is not an individualized approach because dosage adjustments are based on algorithms derived from population-level data (Holford et al., 2020). A more individualized approach that is adapted throughout the course of illness is model-informed precision dosing (MIPD) (Abdul-Aziz et al., 2020; Holford et al., 2020; Fratoni et al., 2021). The three principles of MIPD of drugs are: 1) define a specific drug exposure target that reflects the risk-benefit profile for the population but also individualized to the patient, 2) combine population-level information (population PK models) with individual patient factors (e.g., weight, kidney function) to predict doses required to achieve the target, 3) apply an interventional method that uses Bayesian principles and measured drug concentrations to propose dosing regimens expected to achieve the target (Supplemental Figure S1) (Abdul-Aziz et al., 2020; Holford et al., 2020; Fratoni et al., 2021). MIPD is used iteratively and is constantly adapted as the drug PK parameters (e.g., volume of distribution, clearance) change throughout the patient’s dynamic illness course, making it distinctly different from traditional TDM.

MIPD improves outcomes for many drugs and is used in clinical practice in some expert centers (van Lent-Evers et al., 1999; Mizuno et al., 2017; Neely et al., 2018; McGann et al., 2019; Vinks et al., 2019; Mizuno et al., 2020; Rybak et al., 2020; Xiong et al., 2021). In the most recent vancomycin dosing guidelines, (Rybak et al., 2020), MIPD is recommended as it not only improves probability of target attainment but also reduces nephrotoxicity (Neely et al., 2018). Similarly, MIPD improves efficacy of aminoglycosides and reduces incidence of aminoglycoside nephrotoxicity (van Lent-Evers et al., 1999). In our institution, MIPD is used for immunosuppressants (Mizuno et al., 2017) and for hydroxyurea in patients with sickle cell anemia, (McGann et al., 2019), all with improved outcomes. We have incorporated decision support tools for MIPD into dashboards within electronic health records (EHRs) for morphine in neonates (Vinks et al., 2019) and infliximab in children with inflammatory bowel disease (Xiong et al., 2021). MIPD has strong potential to improve efficacy and decrease toxicity of antibiotics. Therefore, in this review, we will discuss the knowledge gaps that remain not just for implementation of β-lactam TDM, but also of MIPD in children.

## Who might benefit from β-lactam precision dosing?

One of the major barriers in implementing MIPD of β-lactam antibiotics in both adults and children is knowing the specific patient subpopulations that would benefit from precision dosing. Patients admitted to the intensive care unit (ICU) have deranged physiology and drug PK/PD alterations can be significant. However, critically ill patients represent a heterogeneous population. Implementation of MIPD for every ICU patient may be resource-intensive and costly. However, simply empirically adjusting antibiotic doses or administration frequency without data-driven management can be dangerous as it carries risks for suboptimal antibacterial activity and antibiotic-associated toxicity (Imani et al., 2017). Broadening antibiotic coverage due to concern for antibiotic failure without confirming PD target attainment can lead to resistance (Jacoby et al., 2010; Meyer et al., 2010; Timsit et al., 2019). Therefore, it is critical to optimize the benefit-to-risk ratio of therapeutic interventions for individual patients, a fundamental concept of precision medicine.

### Patient and clinical factors as identified from population PK models

Population PK models in pediatrics can provide insight into who may benefit from precision dosing through identification of factors contributing to higher- or lower-than average antibiotic clearance or volume of distribution. Patients with higher antibiotic clearance are at risk of low exposures and suboptimal antibacterial efficacy. In Table 1, we summarize several population PK models in critically ill children for ceftriaxone, piperacillin/tazobactam, cefepime and meropenem, the β-lactam antibiotics often used for sepsis in the pediatric ICU (PICU) (Li et al., 2013; Cies et al., 2014a; Nichols et al., 2016; Shoji et al., 2016; Cies et al., 2017; De Cock et al., 2017; Beranger et al., 2019; Al-Shaer et al., 2020b; Hartman et al., 2021; Saito et al., 2021; Tang Girdwood et al., 2021; Yonwises et al., 2021; de Cacqueray et al., 2022). The one patient factor that consistently demonstrates an effect on clearance is estimated glomerular filtration, typically based on creatinine. In some states of critical illness, there is an initial increase in cardiac output, resulting in augmented renal clearance (ARC), up to 3-fold higher than normal (Udy et al., 2011; Udy et al., 2012; Droege et al., 2016; Abdul-Aziz et al., 2020). With higher renal clearance, drugs that are primarily renally excreted, such as β-lactam antibiotics, can be eliminated at faster rates. ARC is a known phenomenon that occurs in critically ill children with increasing age as a risk factor (Rhoney et al., 2021); there are studies that have shown that increased dosing may be needed for antibiotics, including amoxicillin/clavulanic acid, in critically ill children with ARC (De Cock et al., 2015; Van Der Heggen et al., 2019; Dhont et al., 2020). Thus, critically ill children with ARC may be a group to target for precision dosing. It is crucial to identify these patients early in critical illness to ensure adequate antimicrobial coverage.

**TABLE 1:** Summary of patient factors that increase or decrease clearance of ceftriaxone, piperacillin, cefepime, and meropenem as identified by select population pharmacokinetic models in critically ill children.

	Description of critically Ill population (i.e. admitted to intensive care unit)	Patient factors that increase clearance (i.e. higher risk of low exposures and inefficacy)	Patient factors that decrease clearance (i.e. higher risk of high exposures and toxicity)
**Ceftriaxone**			
Hartman, *Clinical Pharmacokinetics*, 2021; 2 compartment model	45 patients aged 0–18 years. Most common dosing regimen: 100 mg/kg once daily, infused for 30 min	High eGFR (>80 ml/min/1.73 m^2^)	Low eGFR
Tang Girdwood, *Antimicrobial Agents and Chemotherapy*, 2022; 2 compartment model	184 patients aged 0–30 years. Most common dosing regimen: 50 mg/kg twice daily, infused for 30 min	High eGFR (>150 ml/min/1.73m^2^). Presence of Fever	Low eGFR. Immature clearance and maturation (from birth to 2 years of age)
**Piperacillin (/Tazobactam)**			
Li, *European Journal of Clinical Pharmacology*, 2013; 2 compartment model	71 patients aged 0–0.15 years (1–56 days). Most common dosing regimen: 100 mg/kg every 8–12 h, infused for 5 min	No significant factor	Immature clearance and maturation (from birth to 2 years of age)
Cies, *Pediatric Infectious Diseases Journal*, 2014; 2 compartment model	13 patients aged 0.75–6 years. Most common dosing regimen: 100 mg/kg every 6 h, infused for 30 min	No significant factor	No significant factor
Nichols, *Antimicrobial Agents and Chemotherapy*, 2016; 1 compartment model	12 patients aged 0.75–11 years. Most common dosing regimen: 100 mg/kg (piperacillin component) every 8 h, infused for 4 h	No significant factor	No significant factor
DeCock, *Journal of Antimicrobial Chemotherapy*, 2017; 2 compartment model	47 patients aged 0–15 years. Most common dosing regimen: 75 mg/kg (piperacillin component) every 6 h, infused for 5–30 min	No significant factor	Immature clearance and maturation (from birth to 2 years of age)
Béranger, *Clinical Pharmacokinetics*, 2018; 1 compartment model	50 patients aged 0–18 years. Most common dosing regimen: 75 mg/kg (piperacillin component) every 6 h, infused for 30 min	High eGFR (>142 ml/min/1.73 m^2^)	Low eGFR
**Cefepime**			
Shoji, *Antimicrobial Agents and Chemotherapy*, 2016; 2 compartment model	91 patients (at least half admitted to ICU) aged 0–16 years. Most common dosing regimen: 50 mg/kg every 8–12 h, infused for 30 min	Low creatinine (i.e., high eGFR)	Immature clearance and maturation (from birth to 2 years of age). High creatinine (i.e., low eGFR)
Al-Shaer, *Antimicrobial Agents and Chemotherapy*, 2020; 2 compartment model	36 patients aged 0–18 years. Most common dosing regimen: 50 mg/kg every 8 h, infused for 30 min	High CrCl (>80 ml/min/1.73 m^2^)	Low CrCl (<80 ml/min/1.73 m^2^)
de Cacqueray, *Clinical Microbiology and Infection*, 2022; 1 compartment model	59 patients aged 0–18 years. Most common dosing regimen: variable	High eGFR (>153 ml/min/1.73m^2^)	Low eGFR (<153 ml/min/1.73m^2^)
**Meropenem**			
Cies, *Journal of Pediatric Pharmacology and Therapeutics*, 2017; 2 compartment model	9 patients aged 1–9 years. Most common dosing regimen: 20 mg/kg every 6–8 h, infused for 30 min	No significant factor	No significant factor
Saito, *Antimicrobial Agents and Chemotherapy*, 2021; 2 compartment models	34 patients aged 0–15 years. Most common dosing regimen: 100 mg/kg/day, 1 h infusions	High eGFR (>35 ml/min—not normalized to body surface area). Meeting 2 or more systemic inflammatory response syndrome (SIRS) criteria	Low eGFR (>35 ml/min—not normalized to body surface area)
Yonwises, *International Journal of Infectious Diseases*, 2021; 1 compartment model	35 patients aged 0–2 years. Most common dosing regimen: 20–40 mg/kg every 8 h, infused for 1 or 3 h	High CrCl (>112 ml/min/1.73 m^2^)	Low CrCl (<112 ml/min/1.73 m^2^)

eGFR, estimated glomerular filtration rate; ICU, intensive care unit; CrCl, creatinine clearance.

Interestingly, meeting two systemic inflammatory response syndrome (SIRS) criteria had an effect of increasing clearance of meropenem in Saito’s model (Saito et al., 2021). SIRS criteria are often met through presence of fever and tachycardia, especially in the setting of infection, in critically ill children. Our model of ceftriaxone in critically ill children also showed that fever increased ceftriaxone clearance (Tang Girdwood et al., 2021). Fever is not a covariate that is frequently considered in the development of population PK models. However, it is biologically plausible that fever leads to tachycardia and increased cardiac output, resulting in increased drug delivery to the kidneys for clearance. While temperature can change rapidly, especially with use of antipyretics, patients who are persistently febrile and tachycardic despite treatment may be another cohort among critically ill children to consider for precision dosing.

Immature renal or liver clearance due to maturation (i.e., maturation effect) is important to consider especially in young children as maturation effect has been found to be a significant covariate in population PK models of β-lactam antibiotics that include a robust number of children under the age of two (Table 1). Prospective studies of β-lactam PK/PD in neonates, especially those born pre-term, are required to best implement precision dosing in this highly vulnerable population (Fuchs et al., 2017).

Other covariates that consistently affect PK parameters have not been identified. While some population PK models for one specific drug may identify a significant relationship for one covariate, others may not. This discrepancy may be due to difficult in identifying correct covariates in pediatrics as a result of co-linearity between covariates, which can lead to selection bias (Piana et al., 2014).

### Impact of extracorporeal support devices on β-lactam PK/PD

Case reports and studies of β-lactam PK/PD in pediatric patients on extracorporeal support devices may provide insight into additional critically ill subpopulations who would benefit from MIPD. There are few to no data of ceftriaxone or piperacillin PK/PD in pediatric patients on extracorporeal membrane oxygenation (ECMO) and/or continuous renal replacement therapy (CRRT) (Stitt et al., 2022). Case studies from our group using population PK models, observed concentrations, and Bayesian estimation to estimate concentration-time profiles have shown that dosing ceftriaxone every 12 h, which is twice as often as standard practice in most PICUs, is sufficient in ECMO but may lead to ceftriaxone accumulation in CRRT (Cervantes et al., 2022; Contreras et al., 2022). These findings suggest using standard ceftriaxone dosing in ECMO, while considering increasing the interval between ceftriaxone doses during CRRT, and are similar to reported findings in adults (Goto et al., 2016; Cheng et al., 2022). However, with a dearth of data for ceftriaxone in extracorporeal support in children, it is difficult to conclude whether MIPD would be warranted.

For piperacillin, a case series in neonates and infants showed that ECMO increases volume of distribution and that higher dosing of piperacillin/tazobactam may be warranted (Cies and Chopra, 2011; Sutiman et al., 2020). Our group demonstrated that for one patient on Molecular Adsorbent Recirculating System (MARS) coupled with CRRT, the empiric piperacillin/tazobactam dosing regimen did not provide concentrations sufficient to meet stringent PD targets (*f*T>_4xMIC_ for 100% of dosing intervals, 100% *f*T>_4xMIC_) while on MARS/CRRT therapy (Tang Girdwood et al., 2020b). Using a previously published PK model, (De Cock et al., 2017), observed concentrations and Bayesian estimation for modeling and simulation, we suggested that increasing the dose while on MARS therapy would be appropriate while a lower than standard dose (80 mg/kg vs. 100 mg/kg) would suffice during CRRT. However, measuring concentrations would allow for reassurance that targets are met, especially when modalities of extracorporeal support are combined and population-level data are limited.

There are more data on cefepime in children on ECMO and CRRT. In a study of 17 children on ECMO, Zuppa et al. found that cefepime clearance was reduced in children on ECMO as compared to previously reported non-ECMO values, but that >25% of doses failed to attain targets, defined as free concentrations above 15 mg/L for at least 70% of the dosing interval (Zuppa et al., 2019). This population PK model was further enhanced through an external validation study, which ultimately included a total of 33 children on ECMO (Thibault et al., 2022). This latter study suggested that when using an MIC of 8 mg/L, a dose of 50 mg/kg every 8 h would be sufficient to reach the defined targets but dosing intervals could be further adjusted based on creatinine and MICs. For CRRT, a case series of four critically ill children on cefepime demonstrated high variability of clearance and volume of distribution among the patients. While a lower target (100% *f*T>_1xMIC_) was met with ∼50 mg/kg every 6–12-h dosing, the most stringent target (100% *f*T>_4xMIC_) was not, consistent with adult studies of cefepime in CRRT, which show that standard intermittent dosing may not achieve targets when MICs are ≥16 mg/L (Al-Shaer et al., 2021) In addition, it is important to note that these children received atypically high CRRT clearance for ammonia clearance due to liver failure: their mean total effluent rate was ∼6,600 ml/min/1.73 m^2^, compared with the more typically prescribed 2,000–3,000 ml/min/1.73 m^2^ (Sutherland et al., 2014; John et al., 2019). Since liver diseases and inborn errors of metabolism, for which high total effluent rates are generally warranted, comprise <15% of all indications for CRRT initiation, the generalizability of these data to patients receiving lower degrees of CRRT clearance are limited. Given the variability in recommended dosing intervals and concerns for sub-optimal dosing in adults, measuring concentrations for precision dosing may be warranted for patients administered cefepime while on CRRT.

Meropenem has the most abundant PK/PD data in children on ECMO and CRRT. The first case reports of infants on ECMO alone or ECMO with CRRT demonstrated that continuous infusion of meropenem met the target of 100% *f*T>_1xMIC_ (Cies et al., 2014b; Cies et al., 2016). A prospective study of six children with sepsis on ECMO and six children with sepsis on CRRT showed that meropenem clearance on either extracorporeal life support device was not different than that of children not on any device, implying that standard doses of 20 mg/kg every 8 h would suffice (Wang et al., 2021a). This same group later developed a population PK model of meropenem in children with sepsis on ECMO, CRRT or both, and based on stochastic simulation, found that 40 mg/kg every 8 h over a 3-h infusion would have only a 75% probability attaining 100% *f*T>_4xMIC_ (Wang et al., 2021b). A population PK model of vancomycin and meropenem in children on ECMO also suggested that higher doses with extended infusions were necessary for meropenem target attainment (Zylbersztajn et al., 2021). For CRRT, the recommendations on meropenem dosing based on both in human studies and *in silico* simulation studies vary greatly with doses ranging from 40 mg/kg/day to 300 mg/kg/day (Nehus et al., 2014; Cies et al., 2016; Nehus et al., 2016; Rapp et al., 2020; Saito et al., 2020; Saito et al., 2021; Tan et al., 2021; Stitt et al., 2022). Dosing intervals range from every 8–12 h, as intermittent or prolonged infusions of 3–4 h, or continuous infusion. Population PK models investigating the effect of CRRT on PK parameters have had varying results. Rapp et al. found that CRRT had impact on clearance, (Rapp et al., 2020), while Saito et al. (2021) found CRRT as a covariate only for volume. Wang et al. (2021a) found no difference in PK parameters between children on CRRT or ECMO compared to patients receiving neither. Without consensus on optimal dosing regimens of meropenem in patients on extracorporeal support life devices, and with higher inter-individual variability, MIPD in this population may be useful.

### Patients at risk for toxicity

Specific studies of β-lactam antibiotic toxicity may reveal critically ill pediatric populations who could benefit from MIPD. Unfortunately, there are limited data relating β-lactam antibiotic PK exposures and toxicity, especially in children. Retrospective studies in pediatrics have shown an increased risk of nephrotoxicity when piperacillin/tazobactam is given with vancomycin compared to when vancomycin is given with other β-lactam antibiotics (Downes et al., 2017; Buhlinger et al., 2019; Cook et al., 2019). One study showed an increased risk of AKI with administration of piperacillin/tazobactam alone (Joyce et al., 2019). However, with limited availability of β-lactam TDM, only data in adults demonstrating a relationship between piperacillin concentrations and AKI exist to help guide MIPD. Imani et al. found a 50% risk of developing nephrotoxicity with a trough concentration >452 mg/L, which is very high compared to commonly targeted MICs (16 mg/L, breakpoint for *Pseudomonas aeruginosa*) (CLSI, 2018). Pediatric-specific studies relating piperacillin PK and nephrotoxicity are warranted to elucidate patient risk factors that may predispose children to AKI and for MIPD use. Notably, in animal models, piperacillin has been shown to impair tubular secretion of creatinine without causing tubular damage (Pais et al., 2020). A recent prospective study in adults has shown that use piperacillin-tazobactam over cefepime is associated with higher rates AKI as defined by changes in serum creatinine but without concurrent changes in blood urea nitrogen or cystatin C (Miano et al., 2022). Therefore, ideally these pediatric studies would include non-creatinine markers of kidney function such as cystatin C and kidney injury-specific urinary biomarkers such as neutrophil gelatinase-associated lipocalin (NGAL) and kidney injury molecule-1 (KIM-1) to distinguish between pseudo-nephrotoxicity from impaired tubular secretion of creatinine and true kidney injury.

Cefepime-associated neurotoxicity has been abundantly reported in adults with studies demonstrating increased risk of neurotoxicity in patients with underlying neurologic abnormalities and renal dysfunction, often when trough concentrations are above 20–30 mg/L (Lamoth et al., 2010; Huwyler et al., 2017; Payne et al., 2017; Boschung-Pasquier et al., 2020; Lau et al., 2020) A toxicodynamic study recommends using a highest trough threshold of 50 mg/L for precision dosing (Lau et al., 2021). Suspected cefepime-associated neurotoxicity in children is limited to case reports, (Alpay et al., 2004; Landgrave et al., 2012; Guzman-Limon et al., 2017; Shah and Bland, 2021; Hambrick et al., 2022), all in children with renal dysfunction; one case reported a cefepime concentration of >60 mg/L days after cefepime discontinuation and another reported a trough concentration of 80 mg/L when the patient was experiencing neurotoxic symptoms. Larger studies to investigate the relationship between cefepime exposures and neurotoxicity specifically in patients at risk of cefepime-associated neurotoxicity (i.e., patients with abnormal neurologic baseline, altered blood-brain barrier integrity, or renal dysfunction) would help identify pediatric-specific toxicity target thresholds to avoid with precision dosing.

## How does target attainment affect outcomes?

Another barrier to implementation of TDM or MIPD is that while monitoring concentrations and adjusting doses increase probability of attaining targets, it remains unknown how target attainment affects clinical outcomes. One reason for the lack of clarity on effect on outcomes is the lack of consensus of the appropriate PD target for critically ill patients. The minimum target, based on murine models of infections, (Craig, 1998), is defined as free concentrations above MIC for 40%–70% of the dosing interval (40–70% *f*T_>MIC_), with the specific percentage being antibiotic dependent. Data suggest that more stringent targets, including 100% *f*T_>MIC_ and 100% *f*T_>4xMIC_, are needed in immunocompromised patients or critically ill patients for microbiological cure (Tam et al., 2002; McKinnon et al., 2008; Roberts and Lipman, 2009; Zhou et al., 2011; Duszynska et al., 2012; Barreto et al., 2021). In addition, the need for more stringent PD targets may be necessary since concentrations at the site of infection may be lower than plasma concentrations (Roberts et al., 2009a; Roberts et al., 2009b; Felton et al., 2014; Veiga and Paiva, 2018). However, evidence regarding whether clinical outcomes in adults are improved with these more stringent targets is mixed, (Sadaba et al., 2004; McKinnon et al., 2008; Taccone et al., 2010; Zhou et al., 2011; Duszynska et al., 2012; Roberts et al., 2012; Rhodes et al., 2015; Abdul-Aziz et al., 2016; Garg et al., 2020; Barreto et al., 2021), leading to a lack of consensus on the optimal PD target.

Adult studies that have evaluated the relationship between target attainment and outcomes have not shown improved mortality rates with target attainment, but have shown an increased bacterial clearance, suppression of antimicrobial resistance, resolution of symptoms and even shorter lengths of stay in the ICU with target attainment (Al-Shaer et al., 2020a; Abdulla et al., 2020; Hagel et al., 2022). To our knowledge, studies relating target attainment and outcomes in critically ill children are lacking. The paucity of studies are likely from inherent difficulties with conducting pediatric studies, such as low enrollment and retention rates, and small sample sizes especially at single centers (Laughon et al., 2011). Studies requiring additional blood draws to measure concentrations are likely to be most affected by these challenges. These same challenges will exist for randomized controlled trials, the gold standard for studies to determine the effect of interventions on outcomes, and prospective observational studies that rely on innovative trial design methods, such as opportunistic sampling and microsampling, will be needed (Laughon et al., 2011). Alternatively, in institutions where precision dosing may have already been implemented, a time-series analysis to evaluate impact on target attainment and outcomes could be conducted, as has been done for vancomycin (Stocker et al., 2021). Furthermore, with lower mortality rates among critically ill children and high prevalence of culture-negative sepsis, (Hazwani et al., 2020), identifying clinically-relevant outcomes specific to pediatrics is of utmost importance. Given the difficulty to obtain clinical specimens in children, relevant outcomes may include reduction in attaining cultures and laboratory evaluations. Children can also have long-term morbidity effects from severe infections, like sepsis, (Meert et al., 2020; Wong et al., 2021), so longer-term outcomes including need for rehabilitative services should be considered.

## Additional challenges to implementation of β-lactam precision dosing

There are several other challenges to implementation of β-lactam TDM and MIPD that are important to mention. An increasing number of β-lactam/β-lactamase inhibitors are becoming available for use in pediatrics, which present their own complexities for TDM and MIPD. There also exist logistical and financial barriers to implementation of β-lactam TDM and MIPD including development of assays, specifically of unbound concentrations due to variability of protein binding in critical illness (Wong et al., 2013; Wong et al., 2018), whose results can be returned in an appropriate amount of time. Other practical considerations include selecting a precision dosing tool, providing appropriate training, and identifying the personnel best suited for performing MIPD. Ultimately, uptake requires buy-in from the institution to provide the financial investment for these endeavors.

## Discussion

The barriers to implementation of β-lactam TDM and MIPD in critically ill children provide research opportunities to address knowledge gaps. We provide a summary of barriers and potential solutions in Table 2. While the population PK models we selected to review show that estimated GFR by creatinine/creatinine clearance is an influential clinical factor for dosing β-lactam antibiotics, creatinine has several limitations in its ability to reflect true renal function due to tubular secretion causing overestimation of GFR at low GFR levels, unstable renal function in critical illness, and low creatinine production in patients with low muscle mass, (Jelliffe, 2002), leading to inaccurate estimation of renal function. In addition, increases in creatinine lag behind the onset of kidney injury by 24–48 h, and it is possible to sustain tubular injury without changes in creatinine due to the reserve kidney function (Porrini et al., 2019; Armenta et al., 2022; Molitoris, 2022). Therefore, models that incorporate estimates of GFR which are less susceptible to changes in muscle mass, such as cystatin C, and biomarkers of tubular injury such as NGAL, should be developed to identify those patients whose altered renal function could benefit from precision dosing. Cystatin C has been identified as having significant influence on clearance of amoxicillin/clavulanic acid (De Cock et al., 2015). It has also been shown that NGAL can predict changes in the concentration of renally cleared medications such as milrinone prior to changes in serum creatinine (Gist et al., 2018). Until there is more robust evidence to use these biomarkers more uniformly to assess renal function or injury, incorporation of these biomarkers will likely require prospective studies as they are not routinely measured for clinical care at most institutions, except in certain populations with low muscle mass (e.g., bone marrow transplant patients (Benoit et al., 2019)). In addition, systematic inclusion and testing of biologically relevant covariates, such as fever, should be done to identify factors that may predispose patients to low antibiotic exposures and ineffective antibacterial activity. More robust models with larger sample sizes of critically ill children on extracorporeal life support devices are warranted as this subpopulation would likely benefit from MIPD.

**TABLE 2:** Barriers/Challenges and Potential Solutions for Implementation of β-lactam Precision Dosing.

Barriers/Challenges	Potential solutions
Uncertainty of which specific subpopulations among critically ill children would benefit from precision dosing	• Additional studies focused on pre-term and full-term neonates
	• Population PK studies in pediatric patients on extracorporeal support devices
	• Rigorous studies, including physiologically based PK modeling, that relate PK exposures and toxicities
	• Population PK studies that incorporate more specific markers of kidney function including cystatin C and kidney damage including urinary neutrophil gelatinase-associated lipocalin (NGAL)
	• Systematic inclusion and testing of biologically relevant covariates, such as fever
Paucity of data on effect of target attainment and outcomes	• Specific pediatric studies relating target attainment and outcomes
	o Increase enrollment and retention in clinical trials through innovative trial designs, such as use of scavenged opportunistic sampling or microsampling. Laughon et al. (2011)
	o Increase sample size through multi-site collaborations
	• Incorporation of clinically-relevant outcomes specific to pediatrics, including reduction in attaining cultures and laboratory evaluations and long-term morbidity effects
	• Extrapolation from adult studies but recognizing limitations that PK exposures cannot be accurately predicted from models developed in a population with different covariate ranges than population of interest Fuchs et al. (2017)
Complexity introduced by β-lactam/β-lactamase inhibitor combinations	• Dedicated studies to combination drugs
Practical/Logistical Considerations including assay development, selection of precision dosing tool and training of personnel	• Obtaining institutional and provider buy-in about value of MIPD through education of high β-lactam concentration variability and the potential impact on efficacy and toxicity
	• Shared knowledge and resources from institutions who have implemented MIPD of β-lactam antibiotics

With the emerging data of β-lactam associated toxicities, pediatric specific studies to relate antibiotic exposures and toxicity to define toxicity thresholds for MIPD are warranted. This area is also ripe for the development of physiologically-based PK (PBPK) models to estimate antibiotic exposures at sites of toxicity (e.g., kidney, liver, brain) or infection (e.g., lung). Quantitative systems pharmacology (QSP) models can help elucidate the mechanisms of toxicity. Biomarkers of toxicity, and even efficacy (given the lack of consensus on PD targets), is a fertile area for discovery and can be incorporated into QSP models.

As more institutions adopt routine TDM and MIPD for their critically ill patients, including children, it is crucial to disseminate learnings from these services to maximize success at other institutions. Development of clinical decision support tools that are integrated and embedded in the electronic health record or in electronic tertiary resources accessible to multiple healthcare systems can help with implementation and allow for systematic study of how MIPD leads to change in dosing regimens and affects outcomes. It is critical that the data generated from these services are harnessed so that we can identify subpopulations who would most benefit from MIPD and ultimately improve clinical outcomes through precision medicine.
