# Supplementary figures and images for: β-lactam precision dosing in critically ill children: Current state and knowledge gaps
Source: Front Pharmacol. 2022 Dec 1;13:1044683. doi: 10.3389/fphar.2022.1044683 (PMC9752101; doi:10.3389/fphar.2022.1044683)

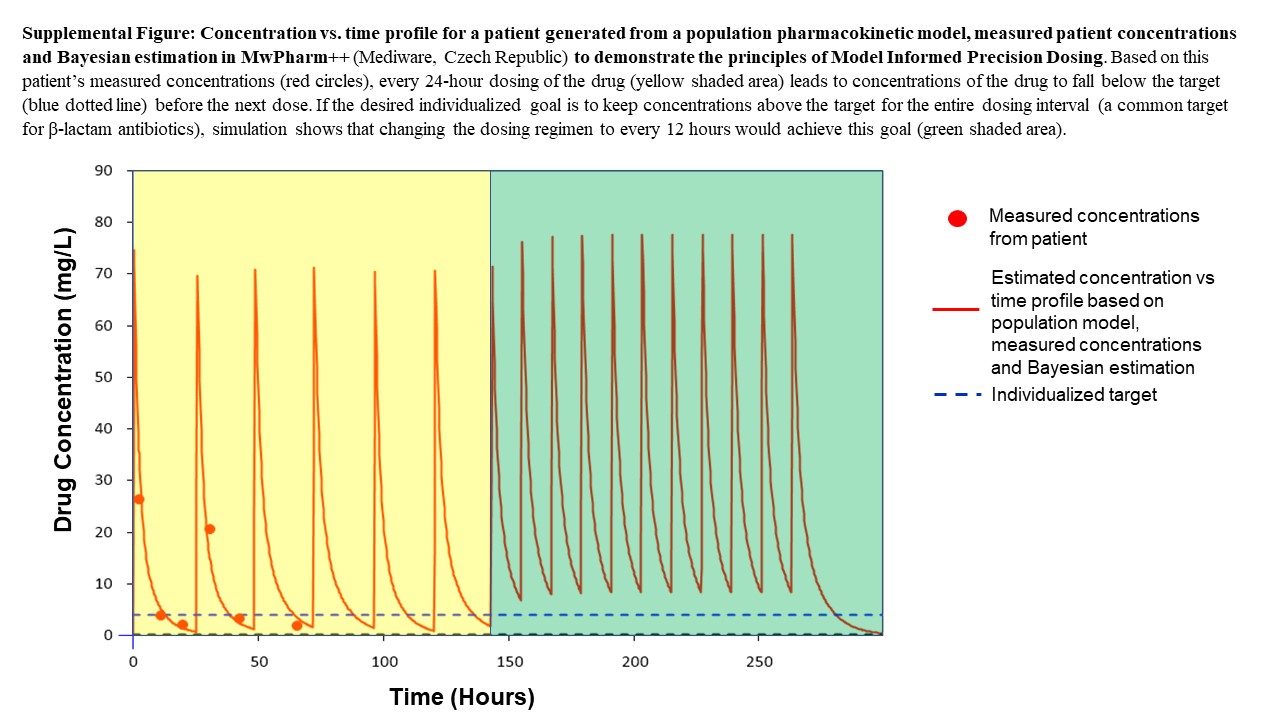

Supplement: Supplementary file 1 [file Image1.JPEG]
